# Supplementary material for: Polygenic Scores for Cognitive Abilities and Their Association with Different Aspects of General Intelligence—A Deep Phenotyping Approach
Source: Mol Neurobiol. 2021 May 5;58(8):4145–56. doi: 10.1007/s12035-021-02398-7 (PMC8280022; doi:10.1007/s12035-021-02398-7)
Supplement: Supplementary file 1 — Supplementary file1 (DOCX 538 KB) [file 12035_2021_2398_MOESM1_ESM.docx]

**Polygenic Scores for Cognitive Abilities and their Association with Different Aspects of General Intelligence – a Deep Phenotyping Approach**

**Genç et al. (2021)**

**Supplemental Material**

**Table S1 Descriptive Statistics for all Cognitive measures.**

|  | **Min** | **Max** | ***M*** | ***SD*** |
| --- | --- | --- | --- | --- |
| **General intelligence**  _(IST-2000-R)_ | 44.00 | 164.00 | 118.83 | 19.67 |
| **Memory**  _(IST-2000-R)_ | 3.00 | 23.00 | 17.07 | 4.06 |
| **Processing speed**  _(ZVT)_ | 32.00 | 95.25 | 54.59 | 10.51 |
| **Matrices**  _(BOMAT)_ | 3.00 | 28.00 | 16.02 | 4.13 |
| **Figural intelligence**  _(IST-2000-R)_ | 12.00 | 55.00 | 35.38 | 8.03 |
| **Numerical intelligence**  _(IST-2000-R)_ | 12.00 | 60.00 | 43.14 | 10.56 |
| **Verbal intelligence**  _(IST-2000-R)_ | 15.00 | 55.00 | 40.31 | 6.15 |
| **General knowledge**  _(IST-2000-R)_ | 15.00 | 80.00 | 53.31 | 9.79 |
| **General knowledge**  _(BOWIT)_ | 61.00 | 295.00 | 145.76 | 39.03 |

**Note.** Table S1 shows descriptive statistics for all cognitive measure raw test scores. Min = minimum test score, Max = maximum test score, *M* = mean test score and *SD* = standard deviation of test score. *N* = 557.

**Table S2** **Intercorrelations between all Raw Test Scores for Cognitive Performance**

|  | **General intelligence**  _(IST-2000-R)_ | **Memory**  _(IST-2000-R)_ | **Processing speed**  _(ZVT)_ | **Matrices**  _(BOMAT)_ | **Figural intelligence**  _(IST-2000-R)_ | **Numerical intelligence**  _(IST-2000-R)_ | **Verbal intelligence**  _(IST-2000-R)_ | **General knowledge**  _(IST-2000-R)_ |
| --- | --- | --- | --- | --- | --- | --- | --- | --- |
| **Memory**  _(IST-2000-R)_ | .395^***^ |  |  |  |  |  |  |  |
| **Processing speed**  _(ZVT)_ | -.571^***^ | -.419^***^ |  |  |  |  |  |  |
| **Matrices**  _(BOMAT)_ | .607^***^ | .442^***^ | -.420^***^ |  |  |  |  |  |
| **Figural intelligence**  _(IST-2000-R)_ | .784^***^ | .338^***^ | -.430^***^ | .522^***^ |  |  |  |  |
| **Numerical intelligence**  _(IST-2000-R)_ | .860^***^ | .312^***^ | -.545^***^ | .521^***^ | .471^***^ |  |  |  |
| **Verbal intelligence**  _(IST-2000-R)_ | .700^***^ | .287^***^ | -.332^***^ | .367^***^ | .396^***^ | .422^***^ |  |  |
| **General knowledge**  _(IST-2000-R)_ | .594^***^ | .162^**^ | -.214^***^ | .331^***^ | .356^***^ | .537^***^ | .514^***^ |  |
| **General knowledge**  _(BOWIT)_ | .408^***^ | .060 | -.051 | .168^***^ | .183^***^ | .359^***^ | .452^***^ | .834^***^ |

**Note.** Table S2 shows the Pearson’s correlation coefficients for the intercorrelations between the test scores of all cognitive measures. *N* = 557, ^***^ *p* ≤ .001, ^**^ *p* ≤ .01, ^*^ *p* ≤ .05.

**Table S3 Intercorrelations between best-fit IQ-PGS**

| **Polygenic score**  **best-fit for…** | **General intelligence**  _(IST-2000-R)_ | **Memory**  _(IST-2000-R)_ | **Processing speed**  _(ZVT)_ | **Matrices**  _(BOMAT)_ | **Figural intelligence**  _(IST-2000-R)_ | **Numerical intelligence**  _(IST-2000-R)_ | **Verbal intelligence**  _(IST-2000-R)_ | **General knowledge**  _(IST-2000-R)_ |
| --- | --- | --- | --- | --- | --- | --- | --- | --- |
|  | PT = .0029 | PT = .0719 | PT = .0037 | PT = .0055 | PT = .0003 | PT = .0055 | PT = .0071 | PT = .0266 |
|  |  |  |  |  |  |  |  |  |
| **Memory**  _(IST-2000-R)_ | .702^***^ |  |  |  |  |  |  |  |
| PT = .0719 |  |  |  |  |  |  |  |  |
| **Processing speed**  _(ZVT)_ | .997^***^ | .727^***^ |  |  |  |  |  |  |
| PT = .0037 |  |  |  |  |  |  |  |  |
| **Matrices**  _(BOMAT)_ | .934^***^ | .753^***^ | .962^***^ |  |  |  |  |  |
| PT = .0055 |  |  |  |  |  |  |  |  |
| **Figural intelligence**  _(IST-2000-R)_ | .814^***^ | .553^***^ | .790^***^ | .755^***^ |  |  |  |  |
| PT = .0003 |  |  |  |  |  |  |  |  |
| **Numerical intelligence**  _(IST-2000-R)_ | .934^***^ | .753^***^ | .962^***^ | 1.000^***^ | .755^***^ |  |  |  |
| PT = .0055 |  |  |  |  |  |  |  |  |
| **Verbal intelligence**  _(IST-2000-R)_ | .908^***^ | .782^***^ | .938^***^ | .973^***^ | .744^***^ | .973^***^ |  |  |
| PT = .0071 |  |  |  |  |  |  |  |  |
| **General knowledge**  _(IST-2000-R)_ | .786^***^ | .912^***^ | .810^***^ | .845^***^ | .635^***^ | .845^***^ | .875^***^ |  |
| PT = .0266 |  |  |  |  |  |  |  |  |
| **General knowledge**  _(BOWIT)_ | .786^***^ | .912^***^ | .810^***^ | .845^***^ | .635^***^ | .845^***^ | .875^***^ | 1.000^***^ |
| PT = .0266 |  |  |  |  |  |  |  |  |

**Note.** To estimate the comparability of the best-fit polygenic scores (PGS) correlation coefficients between each of the scores were calculated. Table S3 shows the Pearson’s correlation coefficient for each PGS pair. PT = *p*-value threshold*. N* = 518, ^***^ *p* ≤ .001, ^**^ *p* ≤ .01, ^*^ *p* ≤ .05.

**Table S4 Intercorrelations between best-fit CP-PGS**

| **Polygenic score**  **best-fit for…** | **General intelligence**  _(IST-2000-R)_ | **Memory**  _(IST-2000-R)_ | **Processing speed**  _(ZVT)_ | **Matrices**  _(BOMAT)_ | **Figural intelligence**  _(IST-2000-R)_ | **Numerical intelligence**  _(IST-2000-R)_ | **Verbal intelligence**  _(IST-2000-R)_ | **General knowledge**  _(IST-2000-R)_ |
| --- | --- | --- | --- | --- | --- | --- | --- | --- |
|  | PT = .0317 | PT = .0119 | PT = .0570 | PT = .0350 | PT = .0001 | PT = .0918 | PT = .0089 | PT = .2030 |
| **Memory**  _(IST-2000-R)_ | .915^***^ |  |  |  |  |  |  |  |
| PT = .0119 |  |  |  |  |  |  |  |  |
| **Processing speed**  _(ZVT)_ | .948^***^ | .861^***^ |  |  |  |  |  |  |
| PT = .0570 |  |  |  |  |  |  |  |  |
| **Matrices**  _(BOMAT)_ | .992^***^ | .906^***^ | .960^***^ |  |  |  |  |  |
| PT = .0350 |  |  |  |  |  |  |  |  |
| **Figural intelligence**  _(IST-2000-R)_ | .581^***^ | .644^***^ | .539^***^ | .567^***^ |  |  |  |  |
| PT = .0001 |  |  |  |  |  |  |  |  |
| **Numerical intelligence**  _(IST-2000-R)_ | .914^***^ | .837^***^ | .958^***^ | .922^***^ | .517^***^ |  |  |  |
| PT = .0918 |  |  |  |  |  |  |  |  |
| **Verbal intelligence**  _(IST-2000-R)_ | .889^***^ | .973^***^ | .830^***^ | .879^***^ | .676^***^ | .805^***^ |  |  |
| PT = .0089 |  |  |  |  |  |  |  |  |
| **General knowledge**  _(IST-2000-R)_ | .842^***^ | .775^***^ | .885^***^ | .850^***^ | .472^***^ | .935^***^ | .751^***^ |  |
| PT = .2030 |  |  |  |  |  |  |  |  |
| **General knowledge**  _(BOWIT)_ | .999^***^ | .914^***^ | .950^***^ | .993^***^ | .578^***^ | .916^***^ | .888^***^ | .846^***^ |
| PT = .0323 |  |  |  |  |  |  |  |  |

**Note.** To estimate the comparability of the best-fit polygenic scores (PGS) correlation coefficients between each of the scores were calculated. Table S4 shows the Pearson’s correlation coefficient for each PGS pair. PT = *p*-value threshold. *N* = 518, ^***^ *p* ≤ .001, ^**^ *p* ≤ .01, ^*^ *p* ≤ .05

**Table S5 Intercorrelations between best-fit EA-PGS**

| **Polygenic score**  **best-fit for…** | **General intelligence**  _(IST-2000-R)_ | **Memory**  _(IST-2000-R)_ | **Processing speed**  _(ZVT)_ | **Matrices**  _(BOMAT)_ | **Figural intelligence**  _(IST-2000-R)_ | **Numerical intelligence**  _(IST-2000-R)_ | **Verbal intelligence**  _(IST-2000-R)_ | **General knowledge**  _(IST-2000-R)_ |
| --- | --- | --- | --- | --- | --- | --- | --- | --- |
|  | PT = 1 | PT = 1 | PT = .1500 | PT = 1 | PT = .1500 | PT = 1 | PT = .2390 | PT = 1 |
|  |  |  |  |  |  |  |  |  |
| **Memory**  _(IST-2000-R)_ | 1.000^***^ |  |  |  |  |  |  |  |
| PT = 1 |  |  |  |  |  |  |  |  |
| **Processing speed**  _(ZVT)_ | .947^***^ | .947^***^ |  |  |  |  |  |  |
| PT = .1500 |  |  |  |  |  |  |  |  |
| **Matrices**  _(BOMAT)_ | 1.000^***^ | 1.000^***^ | .947^***^ |  |  |  |  |  |
| PT = 1 |  |  |  |  |  |  |  |  |
| **Figural intelligence**  _(IST-2000-R)_ | 1.000^***^ | 1.000^***^ | .947^***^ | 1.000^***^ |  |  |  |  |
| PT = .1500 |  |  |  |  |  |  |  |  |
| **Numerical intelligence**  _(IST-2000-R)_ | .968^***^ | .968^***^ | .978^***^ | .968^***^ | .968^***^ |  |  |  |
| PT = 1 |  |  |  |  |  |  |  |  |
| **Verbal intelligence**  _(IST-2000-R)_ | 1.000^***^ | 1.000^***^ | .947^***^ | 1.000^***^ | 1.000^***^ | .968^***^ |  |  |
| PT = .2390 |  |  |  |  |  |  |  |  |
| **General knowledge**  _(IST-2000-R)_ | 1.000^***^ | 1.000^***^ | .947^***^ | 1.000^***^ | 1.000^***^ | .968^***^ | 1.000^***^ |  |
| PT = 1 |  |  |  |  |  |  |  |  |
| **General knowledge**  _(BOWIT)_ | .947^***^ | .947^***^ | 1.000^***^ | .947^***^ | .947^***^ | .978^***^ | .947^***^ | .947^***^ |
| PT = 1 |  |  |  |  |  |  |  |  |

**Note.** To estimate the comparability of the best-fit polygenic scores (PGS) correlation coefficients between each of the scores were calculated. Table S5 shows the Pearson’s correlation coefficient for each PGS pair. PT = *p*-value threshold*. N* = 518, ^***^ *p* ≤ .001, ^**^ *p* ≤ .01, ^*^ *p* ≤ .05.

**
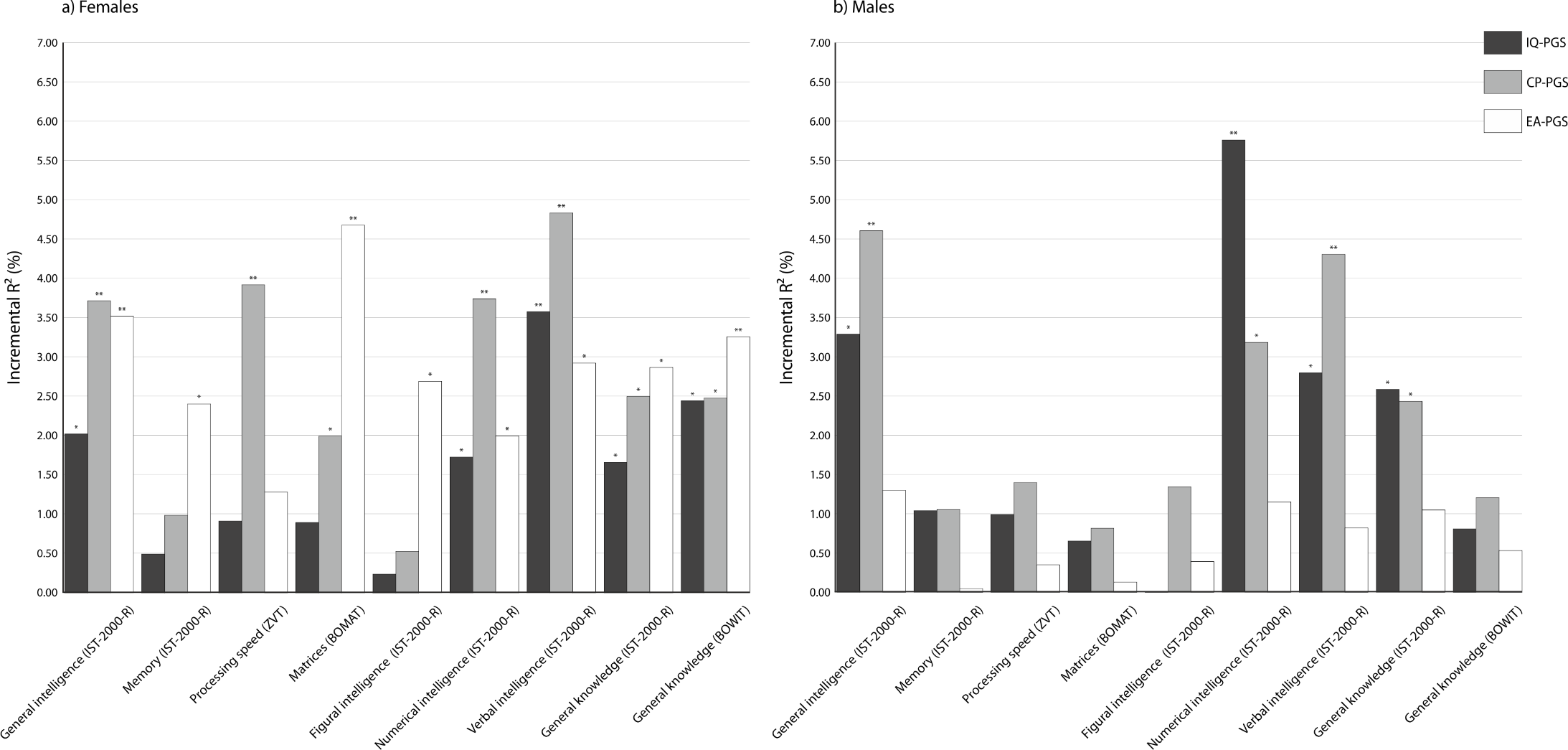
**

**Figure S1.** **Incremental *R*² of the *P*-value threshold (PT) = .05 polygenic scores of intelligence (IQ-PGS), cognitive performance (CP-PGS) and educational attainment (EA-PGS) in percent for females (a) and males (b)**. The incremental *R*^2^ reflects the increase in the determination coefficient (*R*²) when the IQ-PGS or CP-PGS or EA-PGS is added to a regression model predicting individual differences in the respective cognitive test. The association between PGS and phenotype was controlled for the effects of sex, population stratification, and multiple comparisons. ^*^ adjusted *p* ≤ .05, ^**^ adjusted *p* ≤ .01, ^***^ adjusted *p* ≤ .001.

**
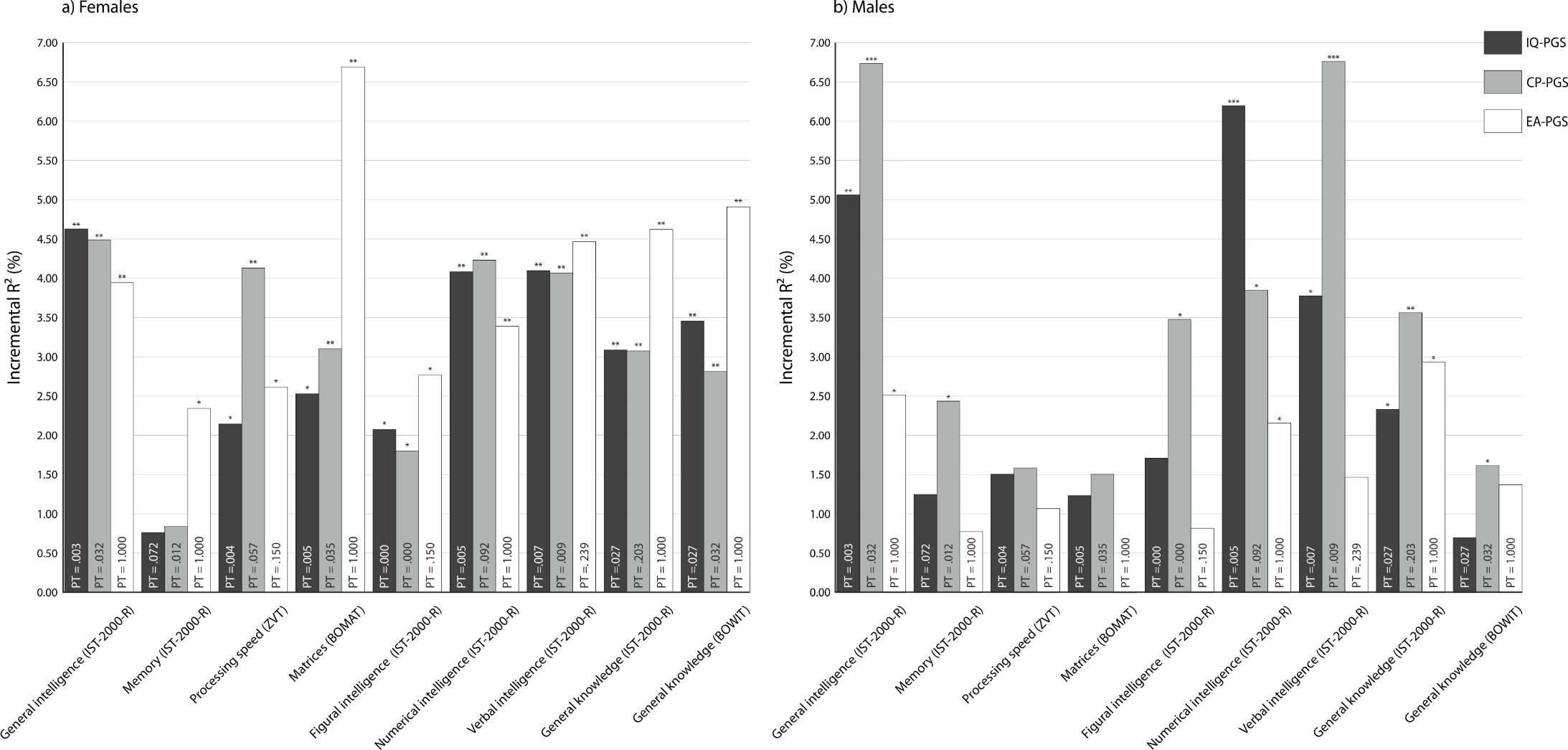
**

**Figure S2.** **Incremental *R*² of the best-fit polygenic scores of intelligence (IQ-PGS), cognitive performance (CP-PGS) and educational attainment (EA-PGS) in percent for females (a) and males (b)**. The *p*-value thresholds (PT) that determined the inclusion of SNPs into the respective PGS are displayed in the respective bar. The incremental *R*^2^ reflects the increase in the determination coefficient (*R*²) when the IQ-PGS or CP-PGS or EA-PGS is added to a regression model predicting individual differences in the respective cognitive test. The association between PGS and phenotype was controlled for the effects of age, population stratification, and multiple comparisons. ^*^ adjusted *p* ≤ .05, ^**^ adjusted *p* ≤ .01, ^***^ adjusted *p* ≤ .001.


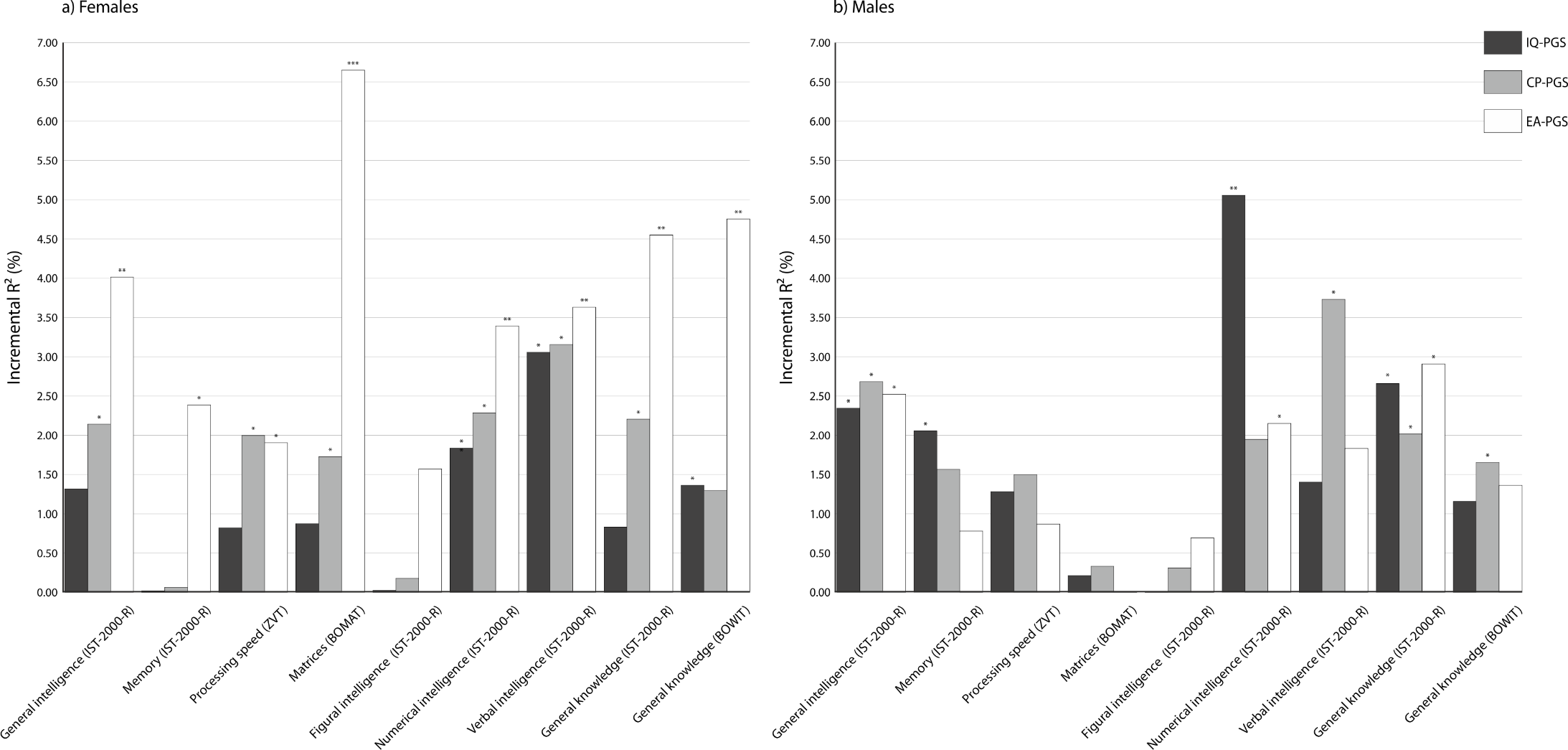


**Figure S3**. **Incremental *R*² of the non-fit (PT = 1) polygenic scores of intelligence (IQ-PGS), cognitive performance (CP-PGS) and educational attainment (EA-PGS) in percent for females (a) and males (b)**. The incremental *R*² reflects the increase in the determination coefficient (*R*²) when the IQ-PGS or CP-PGS or EA-PGS is added to a regression model predicting individual differences in the respective cognitive test. The association between PGS and phenotype was controlled for the effects of age, population stratification, and multiple comparisons. ^*^ adjusted *p* ≤ .05, ^**^ adjusted *p* ≤ .01, ^***^ adjusted *p* ≤ .001.
